# First Chemical Evaluation and Toxicity of *Casinga-cheirosa* to Balb-c Male Mice

**DOI:** 10.3390/molecules19043973

**Published:** 2014-04-02

**Authors:** Dirce M. Estork, Daniela F. Gusmão, Mateus L. B. Paciencia, Ingrit E. C. Díaz, Antonio D. Varella, Riad N. Younes, Luiz F. L. Reis, Edna F. S. Montero, Maria M. Bernardi, Ivana B. Suffredini

**Affiliations:** 1 Graduate Program in Veterinary, Graduate and Research Vice-Dean Office, Paulista University, R. Dr. Bacelar, 1212, Vila Clementino, São Paulo 04026-002, Brazil; E-Mails: dirceestork@gmail.com (D.M.E.); danifgusmão@yahoo.com.br (D.F.G.); marthabernardi@gmail.com (M.M.B.); 2 Center for Research in Biodiversity, Botany Laboratory and Herbarium UNIP and Extraction Laboratory, Paulista University, Av. Paulista, 900, 1° andar, Cerqueira César, São Paulo 01310-100, Brazil; E-Mails: mbpaciencia@hotmail.com (M.L.B.P.); ingrit-uni@hotmail.com (I.E.C.D.); drvarella@yahoo.com.br (A.D.V.); rnyounes@yahoo.com (R.N.Y.); 3 Hospital São José, R. Martiniano de Carvalho, 965, Bela Vista, 1321-001, São Paulo, SP, Brazil; 4 Education and Research Center, Sírio Libanês Hospital, R. Adma Jafet, 91, Bela Vista, São Paulo 01308-000, SP, Brazil; E-Mail: luiz.reis@hsl.org.br; 5 Medicine College, São Paulo University, LIM-62, Av. Dr. Arnaldo, 455, Pinheiros, São Paulo 01246-000, SP, Brazil; E-Mail: edna.montero@gmail.com; 6 Graduate Program in Dentistry, Graduate and Research Vice-Dean Office, Paulista University, R. Dr. Bacelar, 1212, Vila Clementino, São Paulo 04026-002, Brazil

**Keywords:** *Laetia suaveolens*, Salicaceae, toxicity, α-tocopherol, isoquercitrin

## Abstract

*Laetia suaveolens*, known as “casinga-cheirosa”, crude extract EB719 has previously shown cytotoxic activity against prostate cancer and squamous cell carcinoma. For the first time, seven molecules were isolated from its apolar—α-tocopherol (**1**) and sitosterol (**2**)—and polar—3-*O*-caffeoylquinic acid (**3**), 4-*O*-caffeoylquinic acid (**4**), 5-*O*-feruloylquinic acid (**5**), hyperoside (**6**), and isoquercitrin (**7**)—fractions. Acute toxicity was determined in a two-stage experiment: (1) a reduced number of Balb-c male mice received 5000 mg/kg of EB719 to allow evaluation of general activity and other 27 parameters, plus death, up to the establishment of non-lethal dose (NLD), as well as lethal dose 50% (LD_50_); (2) NLD was administered and diazepam introduced as reference drug. EB719 showed LD_50_ = 178.0 mg/kg, and NLD 156.3 mg/kg. In stage one EB719 did not influence general activity, but provoked impairment in grasp reflexes, tail squeeze and breathing; piloerection and cyanosis were increased. In stage two, alterations occurred in auricular reflex, piloerection and breathing after diazepam administration, but not in response to EB719. Intestinal hemorrhage caused by local bleeding was observed after necropsy, and may be the main cause of animals’ death other than a systemic effect of the extract. Although the isolated compounds are biologically and pharmacologically active in both men and animal systems, it is premature to relate their occurrence in EB719 to the observed intestine hemorrhage in mice.

## 1. Introduction

Plants have still played a central role in the treatment of cancer and infectious diseases for the last several decades [1]. The biological activity of antitumor natural products, such as the vinca alkaloids, podophyllotoxin, paclitaxel, camptothecin and others are well described [2] and usually related to targets within the cell cycle. For that reason, normal cells are unavoidably affected by those molecules, which causes one of the biggest problems in cancer chemotherapy which is their side effects.

Brazilian flora is the most rich in the world. For more than 17 years, our group has been collecting plants in the Amazon rainforest and testing them for biological activities [3,4,5]. From the large screening program, *Laetia suaveolens*, popularly known as “casinga-cheirosa” [6,7], was identified as cytotoxic to both prostate [8] and squamous cell carcinomas [9].

The present work aimed to identify the major compounds from the organic extract obtained from the stems and leaves of *L. suaveolens*, here described as EB719. As natural products tend to have important side effects when used in cancer chemotherapy, it is a matter of ethics to determine the toxicity of EB719, using a small number of animals in the first place, in order to provide a concrete basis to support further pharmacological assays in lab animals, as it has never been done before for this particular species.

## 2. Results and Discussion

**Figure 1:**
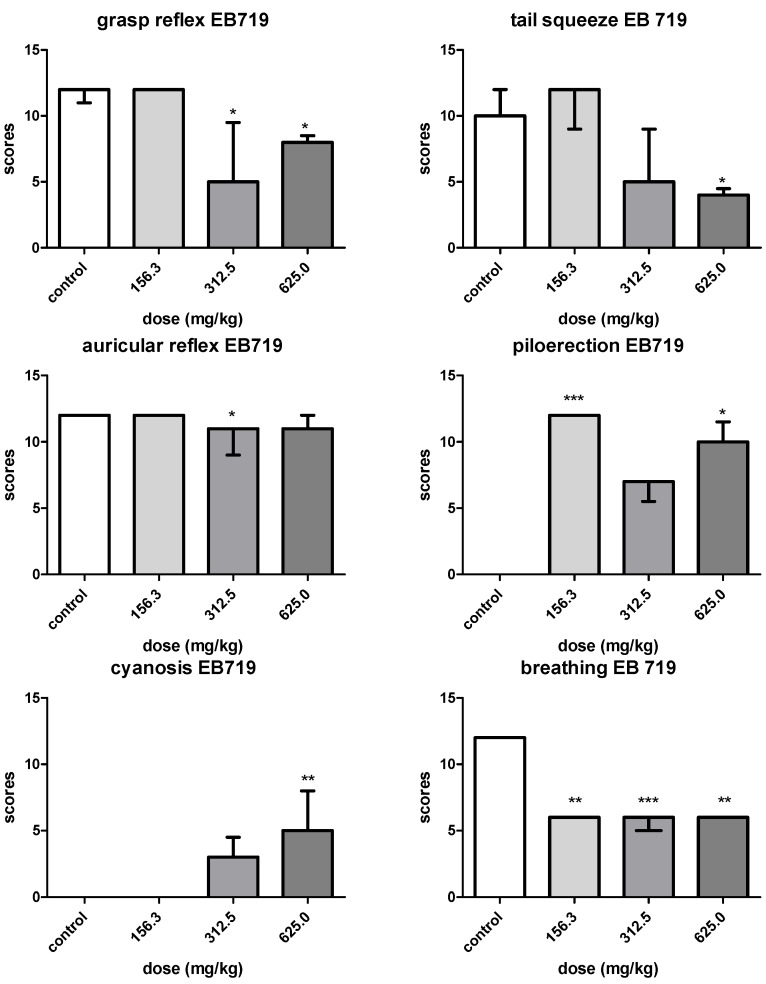
Effect upon murine grasp reflex, tail squeeze, auricular reflex, piloerection, cyanosis and breathing of EB719, obtained from *Laetia suaveolens*, in stage one of the experiment. Kruskall-Wallis statistics (*n* = 3; *N*_total_ = 21) were used for all the parameters. Differences among medians after Dunn’s multiple comparison tests are given and were significant if *p* < 0.05.

The LD_50_ trend was obtained for EB719 using a regression curve and was established as 178.0 mg/kg. According to the parameters established by the European Community, the extract is considered toxic [10]. The administration of EB719 did not influence the general activity of mice. A diminished psychomotor system was observed in the grasp reflex (H~χ^2^_0.05(4)_ = 15.36; *p* < 0.05) after administration of doses of 625.0 and 312.5 mg/kg. A desensibilization of mice in the tail squeeze (H~χ^2^_0.05(4)_ = 11.60; *p* < 0.05) parameter was noted after administration of a dose of 625.0 mg/kg. Alterations in piloerection, auricular reflex, cyanosis and breathing, all related to the autonomous nervous system, were observed after EB 719 administration (Figure 1). Piloerection was pronounced in mice that received doses of 625.0 mg/kg and 156.3 mg/kg (H~χ^2^_0.05,(4)_ = 14.99; *p* < 0.01). Auricular reflexes were diminished after administration of 312.5 mg/kg of EB 719 (H~χ^2^_0.05,(4)_ = 12.71; *p* < 0.05), while cyanosis was observed after administration of 625.0 mg/kg (H~χ^2^_0.05,(4)_ = 15.61; *p* < 0.01). Lastly, breathing was significant diminished after administration of 625.0, 312.5 and 156.3 mg/kg of EB 719 (H~χ^2^_0.05,(4)_ = 17.00; *p* < 0.01).

**Figure 2:**
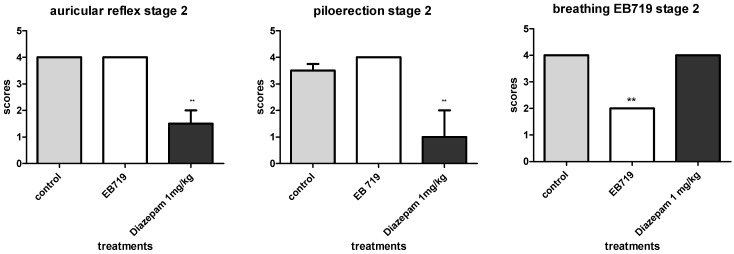
Effect upon murine auricular reflex, piloerection and breathing after administration of the non-lethal dose of EB719, obtained from *Laetia suaveolens*, in stage two of the experiment. Kruskall-Wallis statistics (*n* = 10; *N*_total_ = 30). Differences among medians after Dunn’s multiple comparison tests are given and were significant if *p* < 0.05, when compared to control.

Results obtained from stage two of the experiments (Figure 2) show that diazepam diminished auricular reflex (H~χ^2^_0.05__,(3)_ = 13.36; *p* < 0.01), when compared to both control and EB719 groups, and no differences were observed between control and EB719 groups (*p* > 0.05). Also, piloerection was clearly observed in mice from both control and EB719 groups (H~χ^2^_0.05,(5)_ = 11.70; *p* < 0.01), but mice from the diazepam group showed less piloerection when compared to the EB719 group. Breathing was significantly diminished in the EB719 group (H~χ^2^_0.05,(7)_ = 14.00; *p* < 0.01), but seemed to be normal in both the control and diazepam groups.

**Figure 3:**
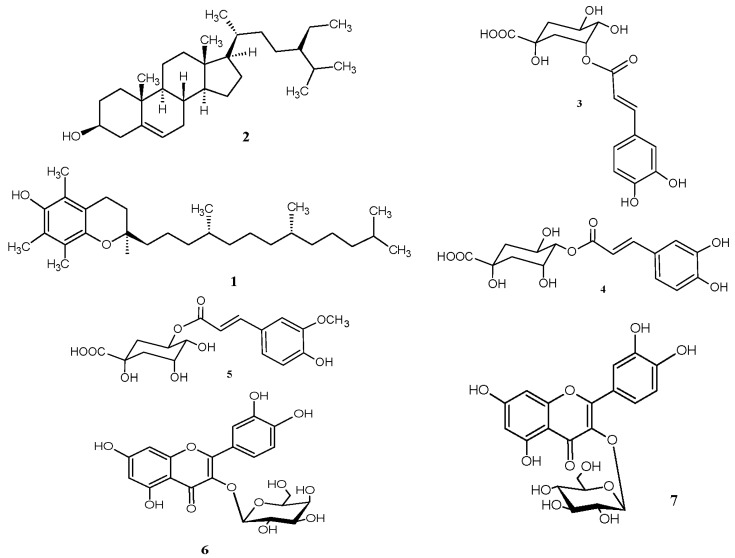
Molecular structures of compounds isolated from leaves and stem of *L. suaveolens* crude organic extract. (**1**) α-tocopherol; (**2**) sitosterol; (**3**) 3-*O*-caffeoylquinic acid; (**4**) 4-*O*-caffeoylquinic acid, (**5**) 5-*O*-feruoylquinic acid; (**6**) hyperoside; (**7**) isoquercitrin.

**Figure 4:**
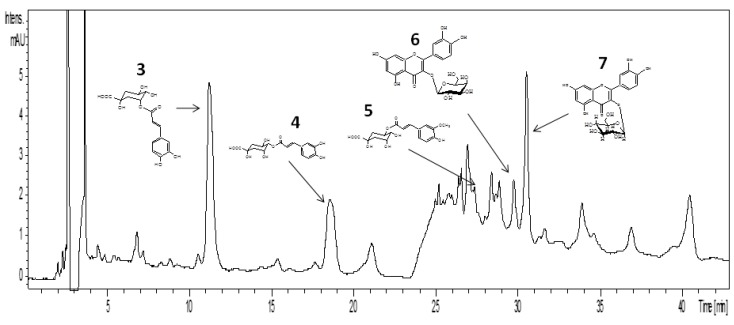
High performance liquid chromatography-UV chromatogram of the crude extract obtained from the leaves and stem of *Laetia suaveolens*, EB719, at λ = 254 nm.

**Figure 5:**
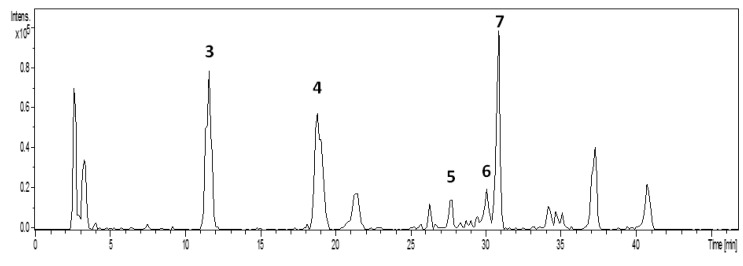
Total ion chromatograms (TIC) of the five compounds in negative ion mode of the crude extract obtained from the leaves and stem of *Laetia suaveolens*, using high performance liquid chromatograpy-(−) ESI-mass spectrometer.

Figure 3 shows the molecular structures of seven compounds isolated from leaves and stem of *L. suaveolens* crude organic extract, and identified as α-tocopherol (**1**) sitosterol (**2**), 3-*O*-caffeoyl-quinic acid (**3**), 4-*O*-caffeoylquinic acid (**4**), 5-*O*-feruoylquinic acid (**5**), hyperoside (**6**) and isoquercitrin (**7**). Figure 4 presents the HPLC-UV chromatogram for the crude extract obtained from the leaves and stem of EB719, obtained at λ = 254 nm. Finally, Figure 5 shows the total ion chromatograms (TIC) in negative ion mode of the five compounds of the crude extract obtained from the leaves and stem of *Laetia suaveolens*, using high performance liquid chromatograpy-(‒) ESI-mass spectrometer. Table 1 shows the retention time, and molecular weight of the five identified compounds that were identified from EB719, obtained from the leaves and stem of *Laetia suaveolens*, by high performance liquid chromatography-mass spectrometry (negative mode).

### Discussion

The search for new pharmacological active compounds from *Laetia* species has previously led to the isolation of clerodane and kaurene diterpenes. Previous studies done with *Laetia* species showed that kaurene diterpenes were isolated from *L. thamnia*, which were tested against colon, prostate and breast cell lines at concentrations ranging from 6 to 50 µg/mL [11]. Also, cytotoxic clerodane esters were isolated from *L. corymbulosa* [12] as well as other six clerodanes were isolated from the trunk bark [13] and were tested against *Plasmodium falciparum* and *Leishmania amazonensis* showing cytotoxicity against the breast cancer cell line MCF-7. Results are in accordance with our initial findings.

**Table 1:** Retention time, molecular weight from the five identified compounds that were identified from EB719, obtained from the leaves and stem of *Laetia suaveolens*, by high performance liquid chromatography-mass spectrometry (negative mode).

Peak No	Substance	Retention Time (min)	Molecular Weight (Da)	[M−H]^−^ *m/z*
3	**3**	11.2–11.8	354	353
4	**4**	18.7–19.1	354	353
5	**5**	27.7	368	367
6	**6**	30.0	464	463
7	**7**	30.8	464	463

The study of the chemical composition of EB719 led to the isolation of compounds 1 to 8, and this represents the first report ever of such molecules in *L. suaveolens*. Compound 1, also known as vitamin E, is frequent mentioned for its antioxidant [14] and anti-inflammatory [15] activities. Previous works show that vitamin E was tested as a radiation protector in people undergoing hemi-body radiation for disseminated disease indicating a likely global effectiveness, although more studies are needed to support the initial findings [16] Vitamin E was also identified as a potent antimutagen when compared to ascorbic acid, *in vitro* [17]. Compound 2 is present in a wide variety of species and has been widely studied worldwide to date. South African scientists found that β-sitosterol and β-sitosterol glucoside enhanced lymphocyte proliferative responses towards phytohaemaglutinin in their cancer patients. So, they performed *in vitro* T-cell proliferative assays with both compounds and phytohaemaglutinin and they observed that a five-fold increase in T-cell proliferation occured [18]. Later, γ-sitosterol isolated from *Markhamia zanzibarica* was found to be cytotoxic to *Artemia salina* [19]. β-Sitosterol is cytotoxic to the PC-3 cell line [20]. Recently, the cytotoxic mechanism of action of γ-sitosterol related to G2/M cell cycle arrest and apoptosis was assessed [21]. The isolated phenolic compounds are also well known, and controversially ambiguous results claiming their carcinogenic or genotoxic potential have already been fully discussed [22,23].

Analysis of acute toxicity was conducted according to previous studies [24], and has already been applied in the analysis of the acute toxicity profile of *Abarema auriculata* [25]. EB719 showed a NLD of 156.3 mg/kg and a LD50 of 178.0 mg/kg, thus being considered toxic. Animal death occurred after administration of the first three higher doses within a period of three hours, and the observations of the 27 parameters were compromised. For that reason, alterations were better interpreted in the groups that received doses of 625.0 mg/kg down to the non-lethal dose (156.3 mg/kg). The first stage of the experiments indicated that the extract interfered with the grasp reflex and with tail squeeze parameters, which may indicate a lack of strength in the forward limbs and that the attention of the mice was directed to some other important occurrences. Alterations related to grasp reflex and tail squeeze were not observed in the second stage of experiment, indicating that the occurrence that influenced both grasp reflex and tail squeeze could not be clearly observed when a NLD was administered. Also, in the first stage of experiments, alterations in sensorial system have occurred in the form of auricular reflex and piloerection, cyanosis and breathing. In the second stage of the experiment, auricular reflex and piloerection still represented significant alterations, despite the administration of a NLD. Piloerection is a parameter that occurs in rodents as a result of fear, illness or pharmacological stress [26]. Still, in the first stage of the experiments, cyanosis and breathing were affected, but only breathing was diminished in the second stage of the experiment. These observations may indicate that the same occurrence affecting the other parameters may have caused diminished breathing and the observation of cyanosis. Parameters related to the central nervous system did not show alterations, in relation to control group.

Necropsy was performed on all experimental mice, either after death during the experiment or at the end of the 14 day observation period. All the animals of the treatment groups showed extensive reddish portions in the small intestine endothelium as well as significant hemorrhages that responded in a dose-dependent way, possibly due to dilated endothelial veins and capillaries and edema. These conditions were present after the administration of all doses causing death, excluding the ones that did not cause death. Bleeding may be the main occurrence that led the animals to die and that caused all the alterations observed in the parameters, both in the first and second stages of experiments. Some plants used as dietary supplements or complementary and alternative medicines, such as Gingko, feverfew, garlic, ginger, ginseng, saw palmetto and willow bark can interfere with clotting processes, altering bleeding time [27], and support our findings related to *L. suaveolens*. d-α-Tocopherol was reported as interfering with prolongation of prothrombin time and partial thromboplastin time in Sprague-Dawley rats, provoking severe hemorrhages in the epididymis and other internal organs [28], although a large number of studies report its use as an antioxidant supplement. No reports describing hemorrhage and sitosterol were found, but increasing concentrations of sitosterol inhibit the proteolytic and phospholipase activities of Brazilian snake venoms [29]. Also, caffeoylquinic acids are said to be antioxidants that can be used in the prevention of hemorrhages, and not to cause hemorrhage [30]. The same observations are made for isoquercitrin [31], which is usually tested as an antioxidant or against snake venoms. No reports relating hyperoside with hemorrhage were found. 

## 3. Experimental

### 3.1. Plant Collection and Extract Preparation

Plant material was collected in the Brazilian Amazon rainforest, under Brazilian Government licenses (license CGen/MMA#12A/2008). The collection was made in the surroundings of Manaus city, in Amazonas state, in a seasonally flooded forest (Igapó forest) in the Rio Negro Basin. The voucher is deposited at UNI.P. Herbarium [A.A. Oliveira, 3383 (UNIP)] and was identified by Mateus L. B. Paciencia, curator of the UNIP Herbarium. Leaves and stem of *L. suaveolens* were processed together and were dried in an air-circulating oven (Fanem, SP, Brazil) at 40 °C and ground in a hammer-mill (Holmes, Danville, IL, USA). Next, 586.10 g of the ground material was placed in a glass percolator (Kontes, Vineland, NJ, USA) and macerated for 24 h with 1.80 L of dichloromethane and methanol (1:1) (Merck, Whitehouse Station, NJ, USA) [2]. Solvents were evaporated under vacuum (Büchi, Flawil, Switzerland) resulting in 43.55 g of crude organic extract, which was maintained in a freezer (Revco, Waltham, MA, USA) until use. Two other collections of the same biological material were made, in order to improve extract amount to be used in the isolation of compounds. Vouchers for the first and second recollections are [M.B.P., 3093 (UNIP)] and [M.B.P., 3203 (UNIP)], respectively. Amounts of 1352.60 g and 2304.45 g were obtained from the dry plant material in the first and second recollections, and resulted in 23.29 g and 21.09 g of crude organic extracts.

### 3.2. Preparation of Extract to Be Administered

Extract EB719 was suspended in almond oil and the following doses were administered: 5000, 2500, 1250, 625, 312.5 and 156.3 mg/kg by intraperitoneal route. Almond oil was used in the extract formulation because of its non polar origin and non-toxic profile, and for that reason, it is compatible as a vehicle for the administration of drugs of natural origin to lab mammals. To avoid alterations of extract composition, the extract suspensions were not sterilized or filtered. The intraperitoneal (I.P.) route was chosen due to the absence of bioavailability loss [25].

### 3.3. Animals

Adult male Balb-C mice (*Mus musculus*) weighing 19–23 g were obtained from the São Paulo University Animals Facilities. After arrival in the laboratory, animals were randomly selected, individually marked, and housed in groups of five in isolated polypropylene cages (38 × 32 × 16 cm), under controlled temperature (21 ± 2 °C) and humidity (55%–60%). Artificial lighting was provided (12 h light/12 h dark cycle, lights on at 8:00 a.m.), as well as free access to Nuvilab^®^ rodent chow (Nuvital Company, São Paulo, Brazil) and an unlimited supply of filtered water. The experiments began one week after the mice arrived, allowing for adaptation to the new laboratory environment conditions. Animals were fasted for a period of one hour before receiving treatments. Animals were observed for toxic responses, and if lethality occurred during the period of observation, a necropsy was performed. On the other hand, if animals survived until the end of the observation period of 14 days, they were humanely submitted to euthanasia in a gas chamber with CO_2_ gas, according to the Ethics Committee directions. Also, the euthanized animals were submitted to necropsy and individual records containing the alterations observed were kept. All experiments were subjected to Ethics Committee protocol (CEP/ICS/UNIP 025/08, approved on 12 February 2009).

### 3.4. Acute Toxicity Signs and Delayed Death Observations

Acute toxicity profiles were assessed [24], with modifications to the number of animals used in the first stage of experiments. Parameters related to general activity, sensory system (such as vocal tremor, irritability, auricular reflex, corneal reflex, tail squeeze, response to touch), psychomotor system (contortion, hindquarter fall, surface-righting reflex, body tone and grasp reflex), central nervous system (convulsions, ataxia, anesthesia, hypnosis, straub tail, tremor, stimulation and sedation) and to autonomic nervous system (lacrimation, breathing, ptosis, piloerection, micturition, defecation, hypothermia and cyanosis) were assessed and a score from 0 to 4 was given for each parameter, considering that 0 is the absence of the effect and 4 is the complete manifestation of the parameter, except to micturition and defecation, which numbers of urination and fecal boli were counted. Body weight for each animal was measured immediately before administration of extract and at least twice during the observation period [25].

### 3.5. Toxicology Experimental Design

A two-stage experiment was designed in order to probe the lethality, general activity, and the toxicity of EB719. In the first stage, the LD50 was determined utilizing three mice per dos. Plus, observations of general activity and toxicity were assessed. The second stage of the experiment was conducted using the non-lethal dose against groups of ten animals each, in which the accuracy of the influence of EB 719 administration over all toxicity parameters could be measured.

In the first stage of the experiments, animals of the control group animals received I.P. administration of almond oil, treatment groups received the corresponding dose of EB719 I.P. Two parameters were obtained from the experiments conducted in the first stage: the LD50 and the NLD. In the second stage of experiment, ten animals were used per extract dose, plus, negative control group received the vehicle and, lastly, diazepam (Hipolabor, MG, Brazil, lot# AO011/11, validity: 10/13, concentration 5 mg/mL, injectable medicine) diluted in sterile H_2_O to 1 mg/kg was used as reference drug in the positive control group. The assays were initiated at 1:00 p.m. and terminated before 5:00 p.m., in order to circumvent circadian influences.

### 3.6. EB719 Dosing for Stage 1 and Stage 2 Toxicity Assays

In stage 1, varying doses of extract were administered, starting at 5,000 mg/kg. If death occurred in at least one of the three treated mice, a half-fold lower dose was administered to a new group of three animals, and observations were repeated. This procedure was repeated until no death was observed. Mice were individually observed in a glass cage for toxic reactions and/or lethality at 10 min, 30 min, 1 h, 2 h and 3 h after administration or until death occurred; mice that survived were observed every 24 h for a 14 additional days, in the event of delayed death. In stage 2, dose was calculated as 10% of the non-lethal dose, obtained in the first stage of the experiment.

### 3.7. Statistical Analysis

In order to organize statistical analysis of non parametric data, the scores (0–4) of each group were summed and formed a new group to be ranked. The Kruskal-Wallis analysis of variance by ranks followed by Dunn post test was then applied. Micturition and defecation were analyzed by one-way ANOVA, followed by Tukey’s multiple comparison tests. All analyses were run under significance level of *p* < 0.05. Statistical procedures were conducted with GraphPad Prism 5.0^®^ and the LD_50_ curve was obtained using GraphPad Instat3.0^®^.

### 3.8. Extract Fractionation and Isolation of Compounds

#### Liquid-Liquid Partition

Organic extract EB719 (5.0 g) was solubilized in methanol (10 mL) and chloroform (5 mL), followed by the addition of water (15 mL). The solubilized extract was transferred to a glass column (2.5 cm intern diameter, 90 cm length). One hundred mL of chloroform were added to the column, and as the chloroform shows a higher density when compared to water, it passes through the polar phase in an intimate contact eluting low polarity substances. The procedure was repeated once more. The chloroform extract was air-evaporated, resulting in a dark brown chloroform residue (RCHCl_3_) (4,025.6 mg; 80.51% yield). From RCHCl_3_, compounds 1 and 2 were isolated and identified. Organic solvent that remained in the aqueous phase was evaporated. The aqueous phase was then subjected to a butanol partition, resulting in the production of a brown butanolic residue (RBuOH) (658.0 mg; 13.16% yield). The aqueous phase was lyophilized (RH_2_O) (316.4 mg; 6.33% yield). RCHCl_3_ was subjected to Sephadex LH20 column chromatography (CC) (2.5 cm intern diameter, 90 cm length) with 300 mL of hexane, 250 mL dichloromethane and 200 mL methanol used to elute the column. This produced FrHEX (3,440.5 mg; 85.47% yield), FrDCM (127.1 mg; 3.16% yield) and FrMeOH (458.0 mg; 11.38% yield). The same fractionation procedure was performed to FrHEX and FrDCM. These were submitted to further fractionation by CC using silica gel (60–200 µm particle size) eluted with mixtures composed of hexane, ethyl acetate and methanol in order of increasing polarity. From these procedures, 50 and 28 fractions were obtained from FrHEX and FrDCM, respectively. All fractions were combined according to analytical thin layer chromatographic (TLC) similarity after visualization with 25% sulfuric acid followed by heating. Sitosterol and α-tocopherol were isolated after purification on silica gel preparative TLC, using a mixture of hexane and ethyl acetate (9:1). 

Organic extracts EB 2093 (23.29 g) and EB 2095 (21.09 g) were combined and also solubilized in methanol and chloroform, followed by the addition of water. Residues (RCHCl_3_), (RBuOH) and (RH_2_O) were obtained once again and were combined to the previous residue obtained from EB 719. Fraction RBuOH was subjected to flash column chromatography (3.5 cm intern diameter and 20 cm length), using 60 g of C-18 silica as stationary phase and eluted with 400 mL of 15% acetonitrile (ACN) in water acidified with 0.1% trifluoroacetic acid (TFA), 500 mL of 50% ACN in 0.1% TFA acidified water and 200 mL of MeOH (0.1% TFA). Three fractions were obtained from RBuOH, named Fr15%ACNRBuOH, Fr50%ACNRBuOH and FrMeOHRBuOH. So, we ended up with larger amounts of each fraction obtained from RBuOH, as follows: Fr15%ACNRBuOH (11.16 g), Fr50%ACNRBuOH (4.08 g) and FrMeOHRBuOH (492.1 mg). Fraction RH_2_O was also subjected to flash column chromatography and fractions Fr10%ACNRH_2_O (9.49 g), Fr50%ACNRH_2_O (2.96 g) and FrMeOHRH_2_O (68.5 mg) were obtained. All fractions were HPLC analyzed in order to obtain fingerprints and sustain their chemical equivalence before combination of samples originated from different collections.

### 3.9. Analytical LC-DAD and Semi-Preparative LC-UV Analysis

Polar fractions were submitted to analytical HPLC using a Shimadzu LC-20 system (Shimadzu, Kyoto, Japan) consisting of an auto sampler, a LC-20AD high-pressure binary pump, a Shimadzu Shimpack ODS HPLC column (5 µm, 250 × 4.6 mm), a series 8042717 oven, and a SPD-M20A photodiode array detector. The HPLC mobile phase conditions were: A = H_2_O (0.1%TFA), B = ACN; 18% B 0–30 min, gradient to 100% B over 45 min, then 100% B for 5 min; flow rate 1 mL/min; injection volume 20 µL; sample concentration 2 mg/mL in MeOH. DAD was set to 210 nm, 254 nm or for full spectra acquisition from 190–800 nm (2 nm resolution). Samples submitted for semi-preparative HPLC were purified using a Shimadzu LC20 system consisting of a LC-6AD series high-pressure pump, a manual injector, a Shimpack-prep ODS HPLC column (5 µm 25 × 2 cm) and a SPD-20A UV detector operated at 254 nm. The column made with Fr15%ACNBuOH was isocratically eluted with 18% ACN in H_2_O (0.1%TFA) and from that elution, compounds 4, 5, 6 and 7 were isolated and identified. The column made with Fr10%ACNH_2_O was isocratically eluted with 10% ACN in H_2_O (0.1% TFA), and from that fraction, compound 3 was isolated and identified Both run conditions were set at 60 min, flow rate 10 mL/min; injection volume = 1 mL; sample concentration = 50 mg/mL dissolved in MeOH; monitored at 254 nm, originating 11 and 10 fractions respectively. From the semi-preparative analysis performed with both fractions, the organic solvent was evaporated from each sample, before being frozen at −70 °C for lyophilization. An amorphous powder was obtained and six compounds (Figure 3) were identified by comparison to existing data from the literature.

α-Tocopherol (**1**): 7.6 mg (0.017% yield) of an orange color resin were isolated and submitted to ^1^H-NMR (500 MHz) and ^13^C-NMR (125 MHz) analysis (see Supplementary Information) to be further compared to existing data [28].

Sitosterol (**2**): 44.0 mg (0.106% yield) was isolated as white crystals and was submitted to ^1^H-NMR (500 MHz) analysis, and ^13^C-NMR (125 MHz) (see Supplementary Information) for comparison with literature data [29].

3-*O*-Caffeoylquinic acid (**3**): 22.5 mg of a cream-colored powder were isolated and submitted to ^1^H-NMR (500 MHz) and to ^13^C-NMR (125 MHz) analysis (see Supplementary Information) [31]; ESI MS (neg. ion mode) *m/z* 352.8 [M−H]^−^.

4-*O*-Caffeoylquinic acid (**4**): 6.3 mg (0.014% yield) of a cream-colored powder were isolated and submitted to ^1^H-NMR (500 MHz) and to ^13^C-NMR (125 MHz) analysis (see Supplementary Information) [31]; ESI MS (neg. ion mode) *m/z* 352.9 [M−H]^−^.

5-*O*-Feruloylquinic acid (**5**): 22.5 mg (0.051% yield) was isolated as a cream-colored powder, and was submitted to ^1^H-NMR and ^13^C-NMR analysis (see Supplementary Information) [30], ESI MS (neg. ion mode) *m/z* 366.8 [M−H]^−^. 

Hyperoside (**6**): 9.6 mg (0.023% yield) of a cream-colored powder were isolated and submitted to ^1^H-NMR (500 MHz) and to ^13^C-NMR (125 MHz) analysis (see Supplementary Information) [32]; ESI MS (neg. ion mode) *m/z* 462.8 [M−H]^−^.

Isoquercitrin (**7**):25.1 mg (0.061% yield) of a cream-colored powder were isolated and submitted to ^1^H-NMR (500 MHz) and to ^13^C-NMR (125 MHz) analysis (see Supplementary Information) [33]; ESI MS (neg. ion mode) *m/z* 462.6 [M−H]^−^. 

### 3.10. Liquid Chromatography—Mass Spectrometry (HPLC/ESI-MS^n^) Analysis

Fractions 15%ACNRBuOH (1.3 mg/mL) and Fr10%ACNH_2_O (1.5 mg/mL) were dissolved in methanol, membrane-filtered (0.45 μm), and analyzed by LC/ESI-MS^n^. A Shimadzu HPLC System consisting of a SCL 10A VP binary pump, a DAD, a SPD MI10A VP, an SIL 10AF auto sampler, and a C18 Luna HPLC column, (5 µm, 4.6 × 250 mm; Phenomenex, Torrance, CA, USA). The column was maintained at room temperature. The mobile phase consisted of A = H_2_O 0.1% HCOOH, B = MeCN; isocratic 18% B; flow rate: 1 mL/min for fraction 15%ACNRBuOH and of A = H_2_O 0.1% HCOOH, B = MeCN; isocratic 10% B; flow rate: 1 mL/min for fraction Fr10%ACNH_2_O.

Crude extract from *L. suaveolens* (1.5 mg/mL) was dissolved in methanol, membrane-filtered (0.45 μm), and analyzed by LC/ESI-MS mode negative. A Shimadzu HPLC System consisting of a SCL Luna HPLC column, (5 μm, 4.6 × 250 mm; Phenomenex). The column was maintained at room temperature. The mobile phase consisted of A = H_2_O 0.1% HCOOH, B = MeCN; flow rate 1 mL/min; gradient 10% B t_0→20min_, 18% B t_20→21min_, 18% B t_21→46min_, 100% B t_46→60min_, 100% B t_60→65min_, 10% B t_65→75min_, 10% B t_75→80min_.

A Bruker Daltonics Esquire 3000 ion trap mass spectrometer (Bruker, Billerica, MA, USA), equipped with electrospray ionization (ESI) source was used. Instrument control and data acquisition were performed using Esquire 5.2 software. The ion source temperature was 320 °C and capillary voltage was set at +4000 V (negative mode) and plat offset −500 V. Nitrogen served as nebulizer gas regulated at 27 psi and a flow rate of 7 L/min. The mass spectrometer was operated in full-scan mode monitoring negative ions. Fragmentation of [M-H]^−^ molecular ions into specific product ions was performed in enhanced product ion (EPI) mode induced by collision with nitrogen.

## 4. Conclusions

Compounds **1** to **8** are reported for the first time to occur in *L. suaveolens*. Although all molecules are biologically or pharmacologically active, no relationship with intestine hemorrhage could be established so far.

## Supplementary Materials

Supplementary materials can be accessed at: http://www.mdpi.com/1420-3049/19/4/3973/s1.
