# Supplementary material for: First Chemical Evaluation and Toxicity of Casinga-cheirosa to Balb-c Male Mice
Source: Molecules. 2014 Apr 2;19(4):3973–87. doi: 10.3390/molecules19043973 (PMC6271492; doi:10.3390/molecules19043973)
Supplement: Supplementary file 1 [file molecules-19-03973-s001.pdf]

Supporting Information

Spectroscopic Data

α-Tocopherol (**1**): $C_{29}H_{50}O_2$. ^{1}H-NMR ($CDCl_3$, 300 MHz) δ: 0,84–0,90 (4CH_3, H-4'a, H-8'a, H-12'a, H-13'), 1.79 (1H, m, H-12'), 2.12 (2CH_3, s, H-5^{a}, H-7a), 2.17 (3H, s, CH_3-8b), 2.62 (2H, t, J = 6.9 Hz, H-4), 4.17 (1H, s, OH); ^{13}C-NMR ($CDCl_3$, 75 MHz) δ: 74.53 (C-2), 23.79 (C-2a), 31.58 (C-3), 20.77 (C-4), 117.36 (C-4a), 118.46 (C-5), 11.25 (C-5a), 144.55 (C-6), 121.01 (C-7), 12.19 (C-7a), 122.62 (C-8), 145.58 (C-8a), 11.75 (C-8b), 39.85 (C-1'), 21.04 (C-2'), 37.43 (C-3'), 32.80 (C-4'), 19.75 (C-4'a), 37.30 (C-5'), 24.45 (C-6'), 37.46 (C-7'), 32.72 (C-8'), 19.75 (C-8'a), 37.49 (C-9'), 24.79 (C-10'), 39.38 (C-11'), 27.98 (C-12'), 22.61 (C-12'a), 22.71 (C-13') The above data were identical to those in the literature data (Matsuo and Urano [1]).

Sitosterol (**2**): $C_{29}H_{50}O$. ^{1}H-NMR ($CDCl_3$, 300 MHz) δ: 3.52 (1H, dddd, J = 9.5 Hz, 4.8 Hz, 11.2 Hz and 4.6 Hz, H-3), 5.36 (1H, s, H-6); ^{13}C-NMR ($CDCl_3$, 75 MHz) δ: 37.25 (C-1), 31.59 (C-2), 71.80 (C-3), 42.24 (C-4), 140.74 (C-5), 121.69 (C-6), 31.91 (C-7), 31.91 (C-8), 50.16 (C-9), 36.50 (C-10), 21.08 (C-11), 39.78 (C-12), 42.32 (C-13), 56.77 (C-14), 24.29 (C-15), 24.29 (C-16), 56.8 (C-17), 11.97 (C-18), 19.37 (C-19), 36.13 (C-20), 19.03 (C-21), 33.96 (C-22), 29.19 (C-23), 50.16 (C-24), 26.13 (C-25), 18.77 (C-26), 19.78 (C-27), 23.08 (C-28), 11.84 (C.29). The data were equal to those of literature data (Holland *et al.* [2]).

Figure S1. HPLC-UV chromatogram of fraction Fr10%ACNRH$_2$O from *Laetia suaveolens*, λ = 254 nm.

D:\Data\CA13\UNIP\Ivana\10-06-13\UNIP-202_1-51_01_282.d\UNIP-202_1-51_01_282.unt
UV (254.0nm)

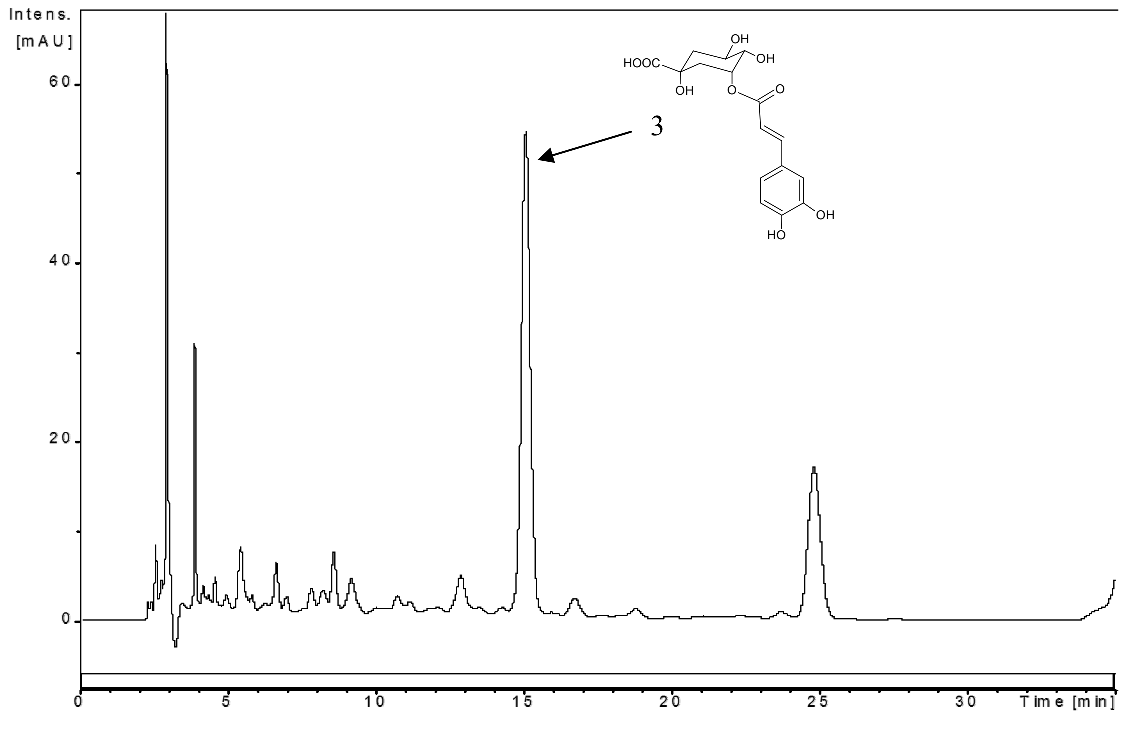

Figure S2. Total ion chromatograms (TIC) of the **3** compound in negative ion mode of fraction Fr10%ACNRH$_2$O from *Laetia suaveolens* from HPLC-(−) ESI-MS.

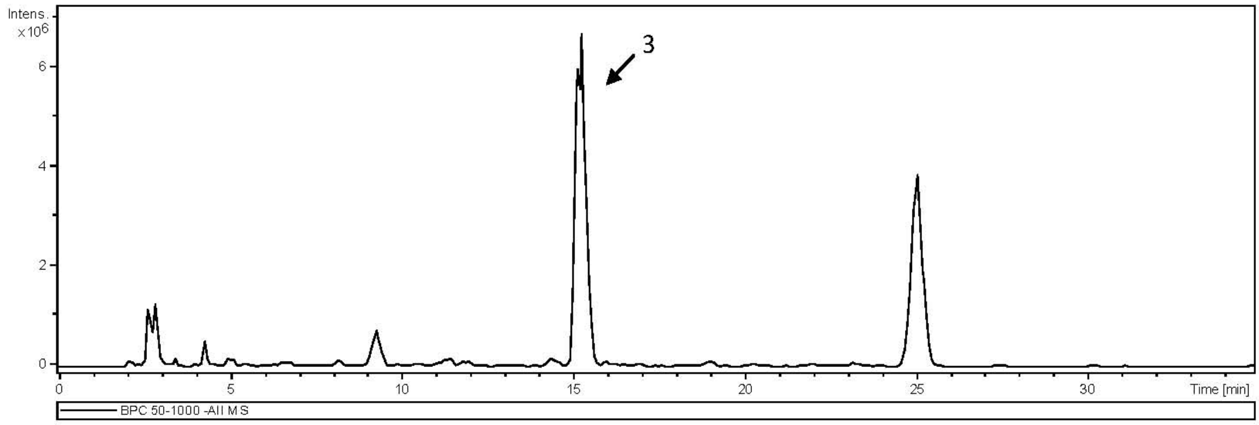

Figure S3. ESI$^-$ mass spectrum of 3-*O*-caffeoylquinico acid (**3**), (2M−H)$^-$ *m/z* 706.8, (M−H)$^-$ *m/z* 352.8 isolated of the fraction Fr10%ACNRH$_2$O from *Laetia suaveloens*.

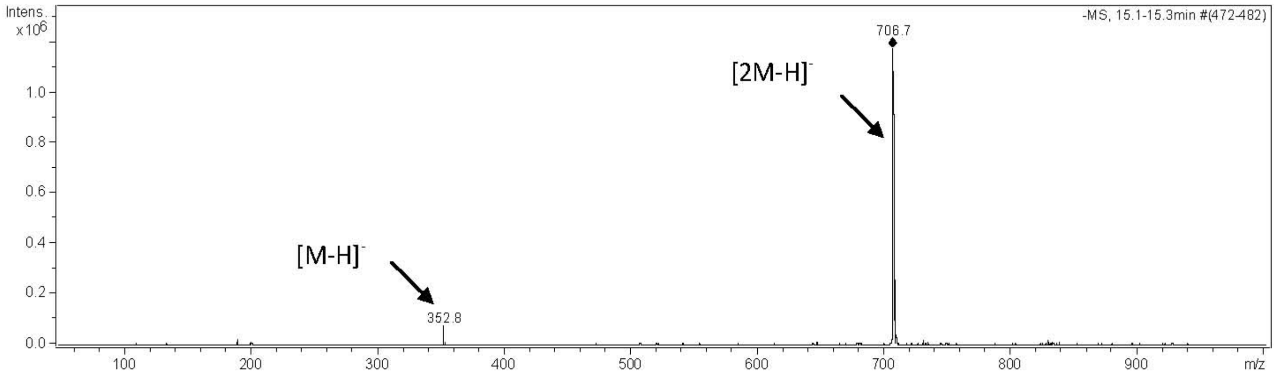

3-*O*-Caffeoylquinico acid (**3**): $C_{16}H_{18}O_9$. ^{1}H-NMR (CD_3OD, 500 MHz) 2.03 (2H, m, H-2$_{eq}$ and H-6$_{ax}$), 2.19 (2H, m, H-2$_{ax}$ and H-6$_{eq}$), 3.69 (1H, m, H-4), 4.16 (1H, s, H-5), 5.36 (1H, m, H-3), 6.77 (1H, d, *J* = 8.15Hz, H-5'), 6.95 (1H, d, *J* = 7.75 Hz, H-6'), 7.05 (1H, s, H-2'), 6.27 (1H, d, *J* = 15.89Hz, H-8'), 7.56 (1H, d, *J* = 16.09 Hz, H-7'); ^{13}C-NMR (CD3OD, 125 MHz) δ: 77.13 (C-1), 35.46 (C-2), 72.43 (C-3), 74.35 (C-4), 67.65 (C-5), 38.66 (C-6), 127.97 (C-1'), 115.31 (C-2'), 146.95 (C-3'), 149.69 (C-4'), 116.64 (C-5'), 123.09 (C-6'), 147.12 (C-7'), 115.55 (C-8'), 169.04 (C-9'), 178.52 (**C**OOH). These data were consistent with those reported in the literature data (Nakatani *et al.* [3]).

Figure S4. HPLC-UV chromatogram of fraction Fr15%ACNRBuOH from *Laetia suaveolens*, λ = 254 nm.

D:\Data\CA13\UNIP\Ivana\10-06-13\UNIP-203_1-52_01_283.d\UNIP-203_1-52_01_283.unt
UV (254.0nm)

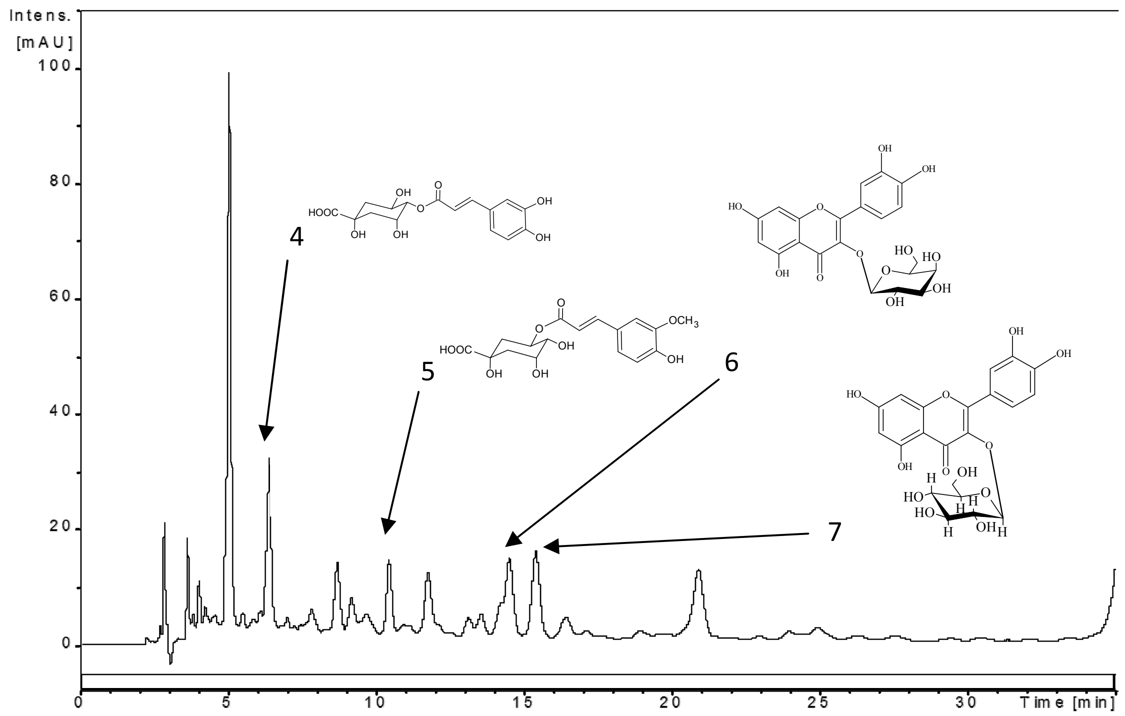

Figure S5. Total ion chromatograms (TIC) of the four compounds in negative ion mode of the fraction Fr15%ACNRBuOH from *Laetia suaveolens* from HPLC-(−) ESI-MS.

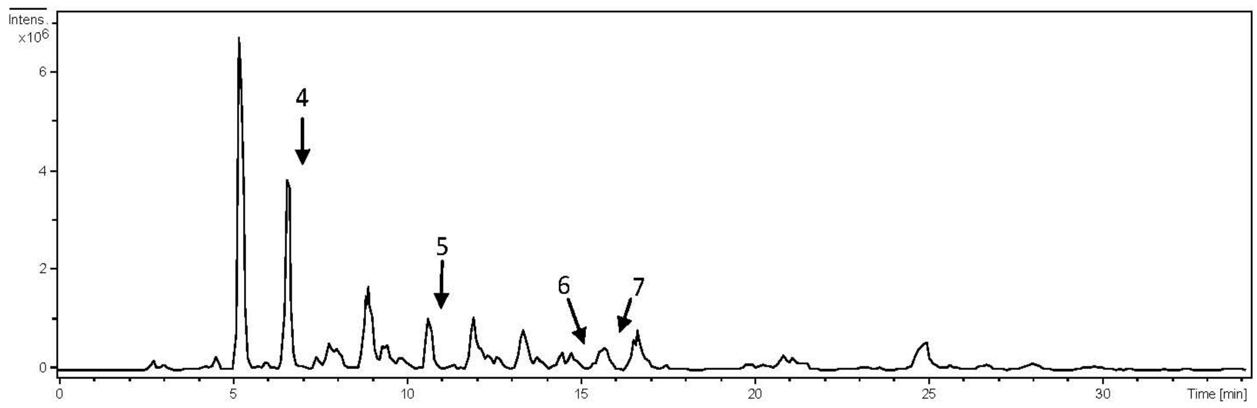

Figure S6. ESI$^-$ mass spectrum of 4-*O*-caffeoylquinico acid (**4**), (2M−H)$^-$ *m/z* 706.7, (M−H)$^-$ *m/z* 352.9 isolated of the fraction Fr15%ACNRBuOH from *Laetia suaveolens*.

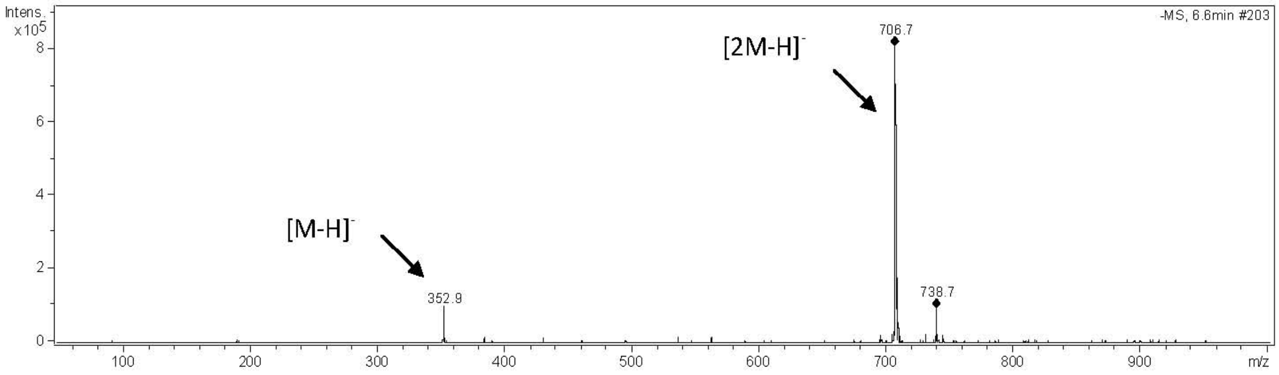

Figure S7. Negative mode MS^2 spectra of [2M−H]$^-$ of 4-*O*-caffeoylquinico acid (**4**).

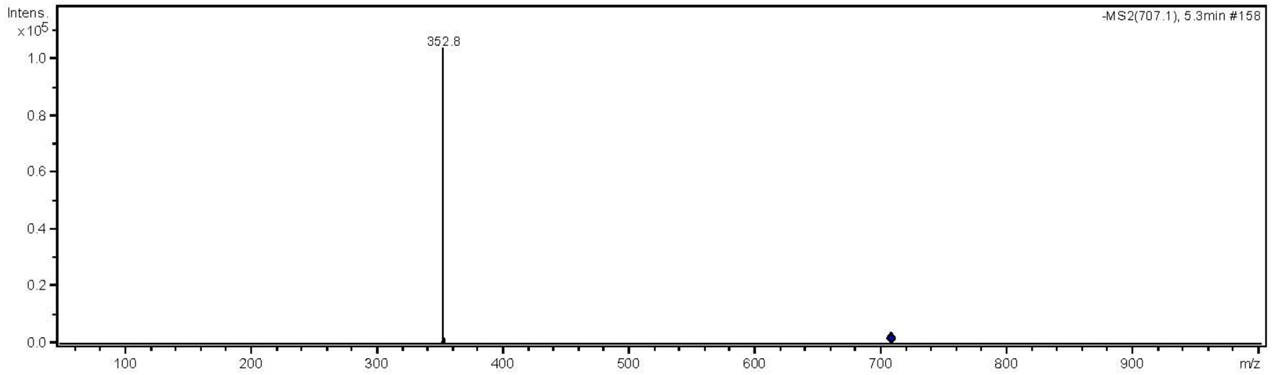

4-*O*-Caffeoylquinico acid (**4**): $C_{16}H_{18}O_9$. ^{1}H-NMR (CD_3OD, 500 MHz) 2.06 (2H, m, H-2_{eq} and H-6_{ax}), 2.19 (2H, m, H-2_{ax} and H-6_{eq}), 3.71 (1H, m, H-5), 4.17 (1H, m, H-3), 5.36 (1H, m, H-4), 6.78 (1H, d, *J* = 8.11 Hz, H-5'), 6.95 (1H, d, *J* = 7.75 Hz, H-6'), 7.05 (1H, s, H-2'), 6.27 (1H, d, *J* = 15.85 Hz, H-7'), 7.56 (1H, d, *J* = 15.97 Hz, H-8'); ^{13}C-NMR (CD3OD, 125 MHz) δ: 76.59 (C-1), 35.92 (C-2), 69.86 (C-3), 78.31 (C-4), 66.56 (C-5), 40.28 (C-6), 127.84 (C-1'), 114.99 (C-2'), 146.98 (C-3'), 149.78 (C-4'), 116.39 (C-5'), 122.76 (C-6'), 147.34 (C-7'), 115.29 (C-8'), 169.31 (C-9'), 176.97 (**C**OOH). Data above is consistent with the literature data (Nakatani *et al.* ([3]).

Figure S8. ESI$^-$ mass spectrum of 5-*O*-feruoylquinic acid (**5**), [2M−H]$^-$ *m/z* 734.7, [M−H]$^-$ *m/z* 366.8 isolated of the fraction Fr15%ACNRBuOH from *Laetia suaveolens*.

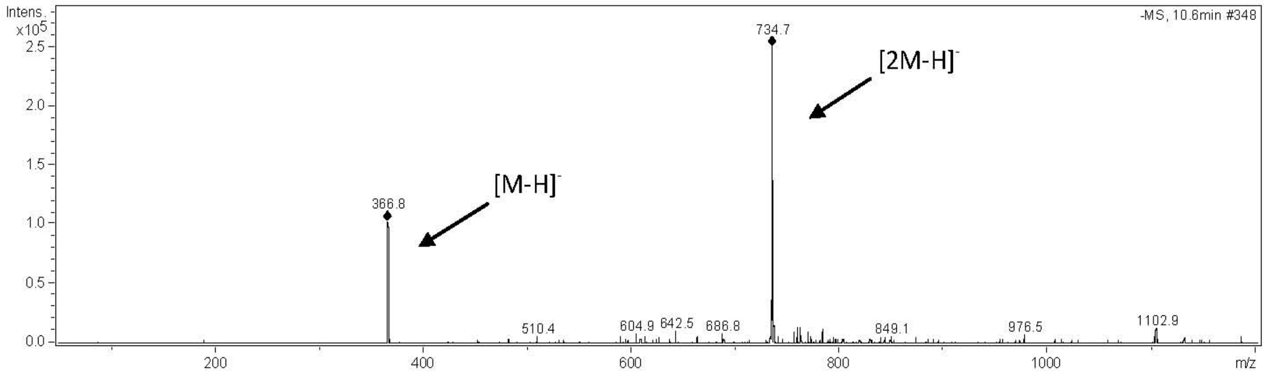

Figure S9. Negative mode MS^2 spectra of $[2M{-}H]^-$ *m/z* 735.1 of 5-*O*-feruoylquinic acid (**5**).

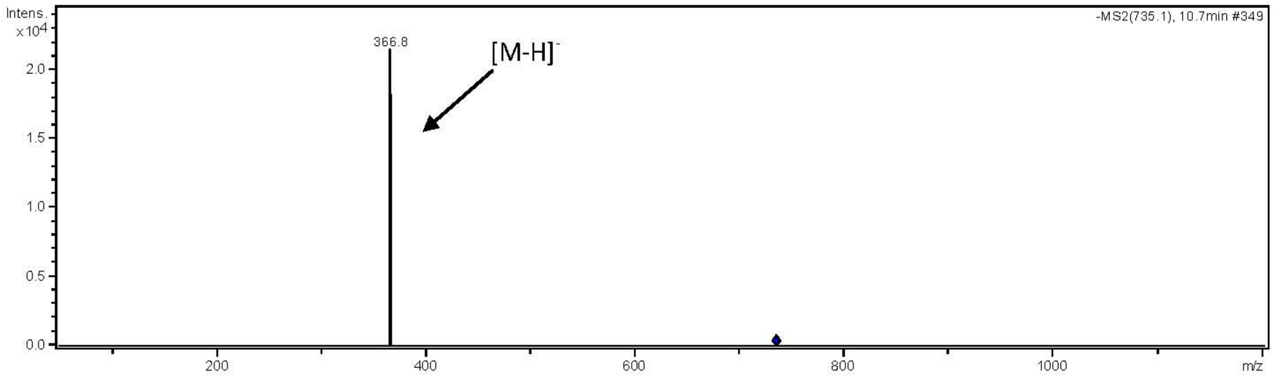

Figure S10. Negative mode MS^2 spectra of $[M{-}H]^-$ *m/z* 366.9 of 5-*O*-feruoylquinic acid (**5**).

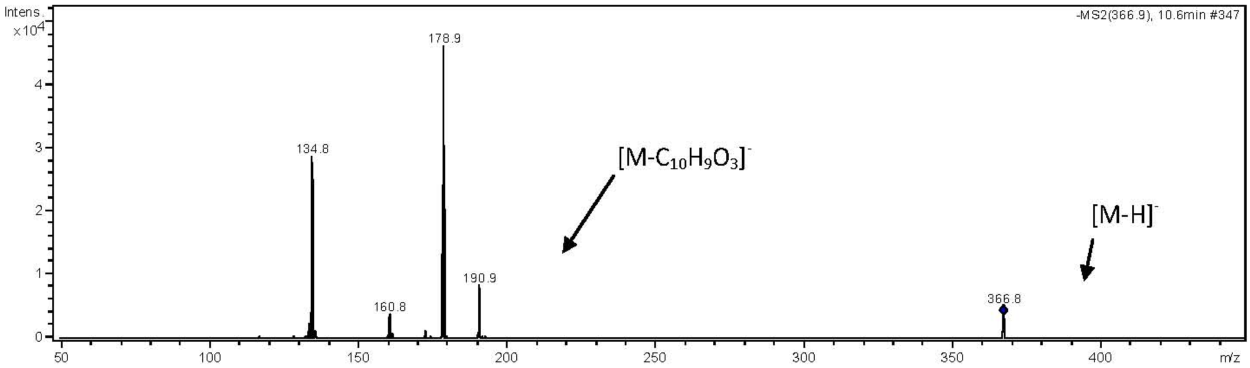

5-*O*-Feruoylquinic acid (**5**): $C_{17}H_{20}O_9$. ^{1}H-NMR (DMSO, 500 MHz) 3.55 (3H, s, H-OCH_3), 6.10 (1H, d, *J* = 15.89 Hz, H-7'), 7.37 (1H, d, *J* = 15.89 Hz, H-8'), 7.023 (1H, d, *J* = 2 Hz, H-2), 6.97 (1H, dd, *J* = 8.15 Hz, 1.99 Hz, H-6), 6.77 (1H, d, *J* = 7.95 Hz, H-5); ^{13}C-NMR (DMSO, 125 MHz) δ: 73.41 (C-1), 33.39 (C-2), 67.63 (C-3), 64.96 (C-4), 71.06 (C-5), 48.66 (C-6), 121.38 (C-1'), 113.91 (C-2'), 165.18 (C-3'), 148.53 (C-4'), 114.59 (C-5'), 116.69 (C-6'), 145.66 (C-7'), 115.86 (C-8'), 161.5 (C-9'), 51.83 (O**C**H_3), 173.57 (**C**OOH). These data were in accordance with those reported in the literature data (Dokli *et al.* [4]).

Figure S11. ESI$^-$ mass spectum of hyperoside (**6**), $[M{-}H]^-$ *m/z* 462.8 isolated of the fraction Fr15%ACNRBuOH from *Laetia suaveolens*.

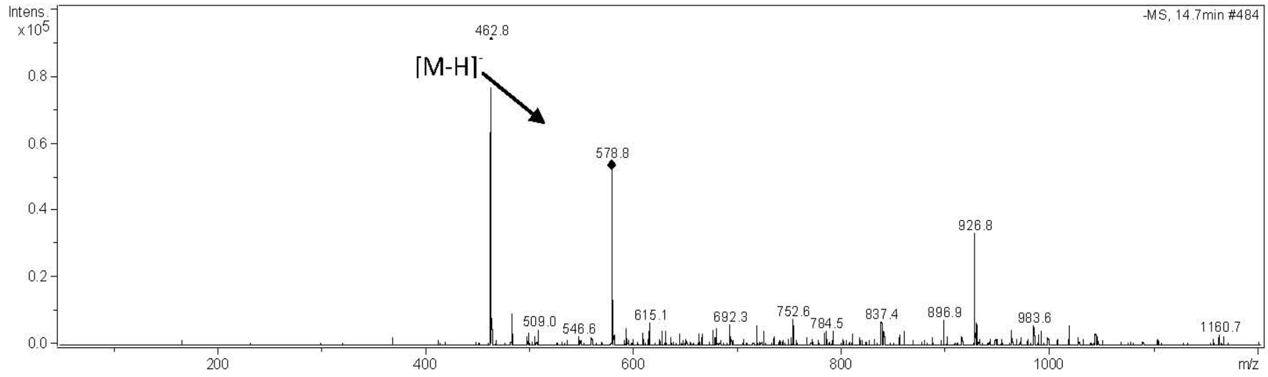

Figure S12. Negative mode MS^2 spectra of $[M-H]^-$ *m/z* 462.9 of hyperoside (**6**).

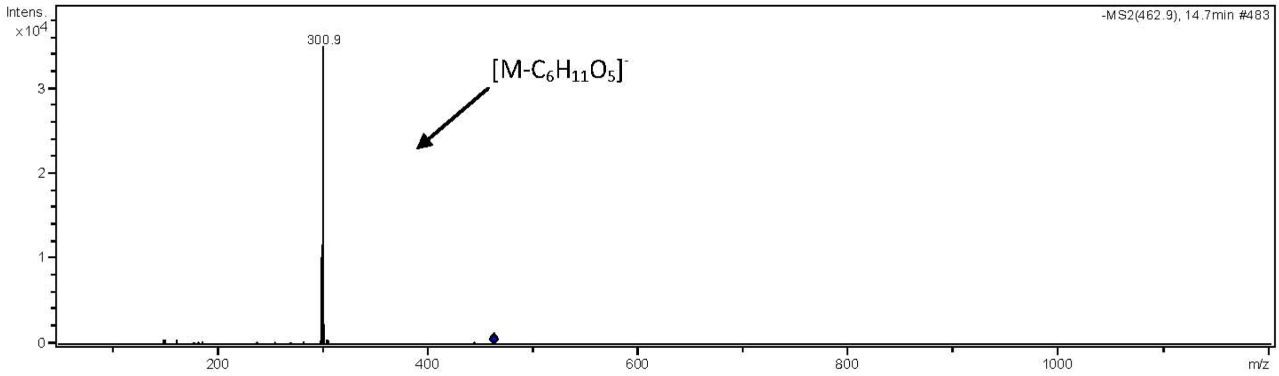

Hyperoside (**6**): $C_{21}H_{20}O_{12}$. ^{1}H-NMR (CD_3OD, 500 MHz) 7.84 (1H, d, *J* = 1.9 Hz, H-2'), 7.59 (1H, dd, *J* = 8.5 Hz, 2.2 Hz, H-6'), 6.87 (1H, d, *J* = 8.5 Hz, H-5'), 6.41 (1H, s, H-8), 6.213 (1H, d, *J* = 1.8 Hz, H-6), 5.17 (1H, d, *J* = 7.9 Hz, H-1''); ^{13}C-NMR (CD3OD, 125 MHz) δ: 158.61 (C-2), 135.49 (C-3), 179.72 (C-4), 163.18 (C-5), 100.05 (C-6), 166.22 (C-7), 94.87 (C-8), 158.65 (C-9), 105.87 (C-10), 123.35 (C-1'), 117.93 (C-2'), 146.08 (C-3'), 150.11 (C-4'), 116.25 (C-5'), 123.10 (C-6'), 105.55 (C-1''), 73.33 (C-2''), 75.25 (C-3''), 70.18 (C-4''), 77.35 (C-5''), 62.10 (C-6''). These data were identical to those in the literature data (Cota *et al.* [5]).

Figure S13. ESI^- mass spectrum of isoquercitrin (**7**), $[M-H]^-$ *m/z* 462.8 isolated of the fraction 15%ACN RBuOH from *Laetia suaveolens*.

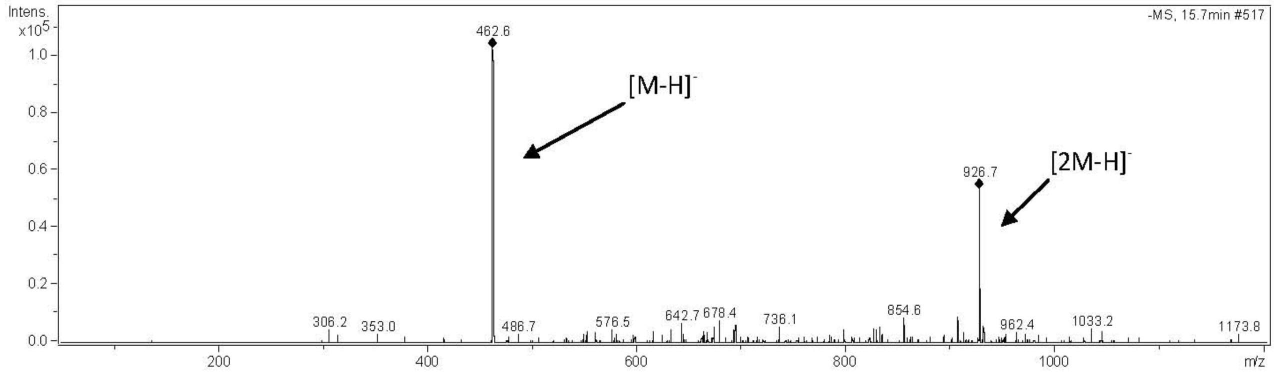

Figure S14. Negative mode MS^2 spectra of $[M-H]^-$ *m/z* 462.7 of isoquercitrin (**7**).

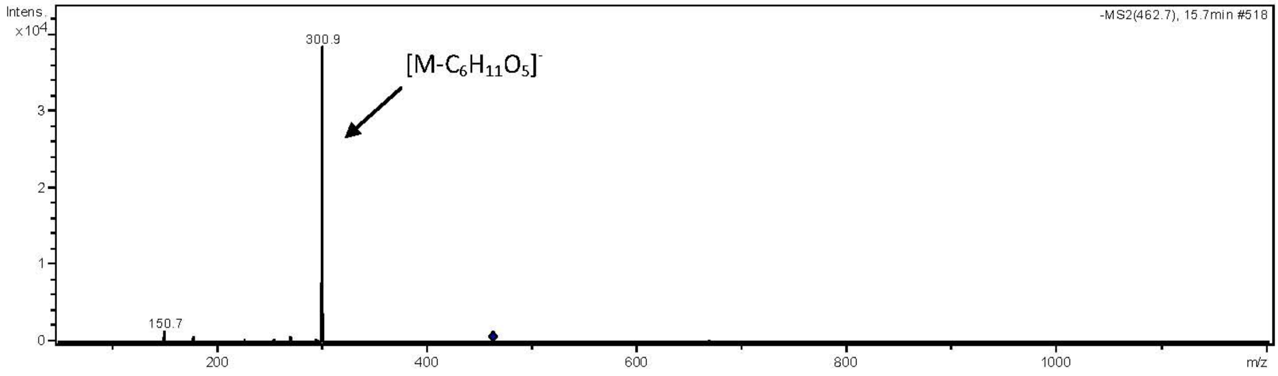

Isoquercitrin (**7**): $C_{21}H_{20}O_{12}$. ^{1}H-NMR (CD_3OD, 500 MHz) 7.71 (1H, d, *J* = 2.4 Hz, H-2'), 7.59 (1H, dd, *J* = 8.5 Hz, 2.2 Hz, H-6'), 6.87 (1H, d, *J* = 8.5 Hz, H-5'), 6.41 (1H, d, *J* = 2.19 Hz, H-8), 6.22 (1H, d, *J* = 1.98 Hz, H-6), 5.26 (1H, d, *J* = 7.76 Hz, H-1''); ^{13}C-NMR (CD_3OD, 125 MHz) δ: 159.19 (C-2), 135.77 (C-3), 179.68 (C-4), 163.24 (C-5), 100.04 (C-6), 166.11 (C-7), 94.85 (C-8), 158.34 (C-9), 105.87 (C-10), 123.24 (C-1'), 117.70 (C-2'), 146.08 (C-3'), 150.02 (C-4'), 116.16 (C-5'), 123.34 (C-6'), 104.43 (C-1''), 75.88 (C-2''), 78.29 (C-3''), 71.39 (C-4''), 78.56 (C-5''), 62.71 (C-6''). These data were consistent with those previously reported in the literature data (Fernandez *et al.* [6]).

References

1. Matsuo, M.; Urano, S. ^{13}C-NMR Spectra of Tocopherols and 2,2-dimethylchromanols. *Tetrahedron* **1976**, *32*, 229–231.
2. Holland, H.L.; Diakow, P.R.P.; Taylor, G.J. ^{13}C Nuclear magnetic resonance spectra of some C-19-hydroxy, C-5,6-epoxy, C-24-ethyl and C-19-norsteroids. *Can. J. Chem.* **1978**, *56*, 3121–3127.
3. Nakatani, N.; Kayano, S.I.; Kikuzaki, H.; Sumino, K.; Katagiri, K.; Mitani, T. Identification, quantitative determination, and antioxidative activities of chlorogenic acid isomers in prune (*Prunus domestica* L.). *J. Agric. Food Chem.* **2000**, *48*, 5512–5516.
4. Dokli, I.; Navarini, L.; Hamersak, Z. Syntheses of 3-,4- and 5-*O*-feruloylquinic acids. *Tetrahedron Asymmetry* **2013**, *24*, 785–790.
5. Cota, B.B.; Siqueira, E.P.; de Oliveira, D.M.; Alves, T.M.A.; Sobral, M.E.G.; Rabello, A.; Zani, C.L. Chemical constituents and leishmanicidal activity from leaves of *Kielmeyera variabilis*. *Rev. Bras. Farmacogn.* **2012**, *22*, 1253–1258.
6. Fernandez, J.; Reyes, R.; Ponce, H.; Oropeza, M.; Vancalsteren, M.-R.; Jankowski, C.; Campos, M.G. Isoquercitrin from *Argemone platyceras* inhibits carbachol and leukotriene D4-induced Concentration in Guinea-pig Airways. *Eur. J. Pharmacol.* **2005**, *522*, 108–115.

© 2014 by the authors; licensee MDPI, Basel, Switzerland. This article is an open access article distributed under the terms and conditions of the Creative Commons Attribution license (http://creativecommons.org/licenses/by/3.0/).
